# Characteristics and Prognosis of Antibody Non-responders With Coronavirus Disease 2019

**DOI:** 10.3389/fmed.2022.813820

**Published:** 2022-06-20

**Authors:** Junyu Ding, Changxin Liu, Zhao Wang, Hua Guo, Kan Zhang, Lin Ma, Bo Wang, Huijun Zhao, Manya Song, Xizhou Guan

**Affiliations:** ^1^Medical School of Chinese PLA, Beijing, China; ^2^Department of Pulmonary and Critical Care Medicine, The Eighth Medical Centre, Chinese People's Liberation Army General Hospital, Beijing, China

**Keywords:** antibody, SARS-CoV-2, COVID-19, infection, immune

## Abstract

**Background:**

Coronavirus disease 2019 (COVID-19), caused by severe acute respiratory syndrome coronavirus 2 (SARS-CoV-2), has been spreading globally. Information regarding the characteristics and prognosis of antibody non-responders to COVID-19 is limited.

**Methods:**

In this retrospective, single-center study, we included all patients with confirmed COVID-19 using real-time reverse transcriptase-polymerase chain reaction (RT-PCR) admitted to the Fire God Mountain hospital from February 3, 2020, to April 14, 2020. A total of 1,921 patients were divided into the antibody-negative (*n* = 94) and antibody-positive (*n* = 1,827) groups, and 1:1 propensity score matching was used to match the two groups.

**Results:**

In the antibody-negative group, 40 patients (42.6%) were men, and 49 (52.1%) were older than 65 years. Cough was the most common symptom in the antibody negative group. White blood cell counts, neutrophils, C-reactive protein, procalcitonin, interleukin-6, lactate dehydrogenase, creatine kinase, creatine kinase isoenzyme, urea nitrogen, and creatinine were significantly higher in the antibody-negative patients than in the antibody-positive group (*P* < 0.005). The number of days of nucleic acid-negative conversion in the antibody-negative group was shorter than that in the antibody-positive group (*P* < 0.001). The hospitalization time of the antibody-negative patients was shorter than that of the antibody-positive patients (*P* < 0.001).

**Conclusion:**

Some COVID-19 patients without specific antibodies had mild symptoms; however, the inflammatory reaction caused by innate clinical immunity was more intense than those associated with antibodies. Non-specific immune responses played an essential role in virus clearance. There was no direct correlation between excessive inflammatory response and adverse outcomes in patients. The risk of reinfection and vaccination strategies for antibody-negative patients need to be further explored.

## Introduction

Since December 2019, a series of unexplained pneumonia cases has occurred in the Wuhan, Hubei Province, China. The pathogen was identified as a novel enveloped RNA beta-coronavirus by gene sequencing and was subsequently named severe acute respiratory syndrome coronavirus 2 (SARS-CoV-2) (1). The World Health Organization (WHO) declared that the disease caused by SARS-CoV-2 was officially named coronavirus disease 2019 (COVID-19) (2). With the rapid spread of COVID-19, as of July 11, 2021, 186,232,998 laboratory-confirmed cases have been documented globally, with 4,027,858 deaths (3).

The treatment protocol for novel coronavirus pneumonia, wherein a diagnostic criterion is that SARS-CoV-2-specific antibodies IgM and IgG are positive, is that IgG antibodies change from negative to positive, or the titer in the convalescent stage has a 4-fold change compared with the acute stage. The host immune response after SARS-CoV-2 infection is vital for evaluating the disease duration and reinfection risk; therefore, understanding patients' immune status is necessary. IgM and IgG have been detected in different studies, revealing that IgM generally shows a trend of rising and then falling, while IgG continues to rise. The positive rate and titer variation of IgG are higher than IgM (4). Studies have also confirmed that IgG antibody levels positively affect virus clearance, while elevated IgM levels indicate poor prognosis in COVID-19 patients (5). However, some studies have found that serum antibodies are not absolutely effective diagnostic targets in some patients, and the severity of coronavirus pneumonia is not entirely associated with the expression of serum antibodies (6). Information regarding the characteristics and prognosis of antibody non-responders with COVID-19 is scarce.

In this study, we performed a retrospective review of the electronic medical records of confirmed COVID-19 patients admitted to the Fire God Mountain hospital to determine antibody non-responders (patients who do not produce specific antibodies against SARS-CoV-2, wherein all antibody tests are negative), and explore the immune mechanism of clearing SARS-CoV-2.

## Materials and Methods

### Study Design and Participants

We retrospectively reviewed the electronic medical records of COVID-19 patients admitted to the Fire God Mountain hospital in Wuhan from February 3, 2020, to April 14, 2020.

All patients with confirmed COVID-19 using real-time reverse transcriptase-polymerase chain reaction (RT-PCR) admitted to the Fire God Mountain hospital from February 3, 2020, to April 14, 2020, were enrolled in the study. The exclusion criteria were as follows: age <18 years, more than 3 months from the initial diagnosis to admission, and no detection of serum IgM/IgG antibodies during hospitalization. We divided 1,921 patients into the antibody-negative group (*n* = 94) and antibody-positive group (*n* = 1,827), and 1:1 propensity score matching (PSM) was used to match the two groups (*n* = 94). Each patient underwent at least one antibody test during the course of disease. When all antibody tests were negative, the patient was considered an antibody non-responder and belonged in the antibody-negative group; when one antibody test was positive, the patient was considered in the antibody-positive group.

According to the Diagnosis and Treatment Protocol for Novel Coronavirus Pneumonia (trial version 7), COVID-19 patients are divided into mild, moderate, severe, and critical cases. Moderate cases showing fever and respiratory symptoms with radiological findings of pneumonia. Severe cases were defined when one of the following criteria was met: (1) shortness of breath, RR ≥ 30 times/min; (2) oxygen saturation is <93% in the resting state; (3) partial pressure of arterial oxygen (PaO_2_)/oxygen concentration (FiO_2_) ≤ 300 mmHg. Critical cases were defined when one of the following criteria was met: (1) respiratory failure; (2) septic shock; and (3) other organ failure. Among these, mild and moderate patients were classified into the general group.

### Ethics Approval and Consent to Participate

All methods used in this study were carried out in accordance with relevant guidelines and regulations. This study was approved by the Ethical Review of Scientific Research Projects of the Medical Ethics Committee of the Chinese PLA General Hospital. The Medical Ethics Committee of the Chinese PLA General Hospital waived the requirement for informed consent. Approval Committee reference number: No. S2020-161-01.

### Data Collection

The data of all patients were obtained using a standardized data collection form from the electronic health records by the research team of the Department of Respiratory, Chinese People's Liberation Army General Hospital. The data collection form included demographic information, date of disease onset, comorbidities, symptoms, signs, laboratory findings, serum antibody test results, and prognosis results.

### Detection of the Antibodies

Serum IgM and IgG antibodies against SARS-CoV-2 were detected using a CFDA-approved chemiluminescence kit by Bioscience Biotechnology Co., Ltd. (Chongqing, China, REF of IgM: C86095M, IgG: C86095G), according to the manufacturer's instructions. A threshold of 10 AU/mL was used for both IgM and IgG as recommended by the manufacturer. A sample threshold of < 10.00 AU/mL was considered non-reactive, and when it was ≥ 10.00 AU/mL, it was considered reactive.

### Statistical Analysis

A 1:1 PSM was used to match the two groups, and the scoring factors were age, sex, hypertension, diabetes, CLD, malignancy, CKD, and COPD. Statistical analyses were performed using Python (version 3.7). We used the mean (SD) to express continuous variables that followed a normal distribution, the median (IQR) to express non-normally distributed values, and the frequency and percentage (%) to indicate categorical variables. Continuous variables were compared using a *t*-test when the variables were normally distributed; otherwise, the Mann-Whitney *U*-test was used. The proportions of categorical variables were compared using the χ^2^ test and prognostic correlation analysis. Logical regression and survival curves were used. Differences were considered statistically significant at *P* < 0.05.

## Results

### Demographic Characteristics

A total of 1,921 laboratory-confirmed COVID-19 patients in the Fire God Mountain Hospital were included in this study, who were divided into the antibody-negative (*n* = 94) and antibody-positive (*n* = 1,827) groups.

The demographic characteristics of the COVID-19 patients are shown in Table 1. In the antibody-negative group, 40 patients (42.6%) were men, and 49 (52.1%) were older than 65 years. There was no significant difference in sex and age between the antibody-negative and antibody-positive groups after matching the two groups with 1:1 PSM.

**Table 1:** Baseline characteristics of patients with COVID-19.

		**Antibody-negative**	**Antibody-positive**	***P***	**Antibody-positive (after matching)**	***P***
*n*		94	1,827		94	
Sex	Female	54 (57.4)	898 (49.2)	0.143	49 (52.1)	0.558
	Male	40 (42.6)	929 (50.8)		45 (47.9)	
Age	<65	45 (47.9)	1,152 (63.1)	0.004	56 (59.6)	0.144
	≥65	49 (52.1)	675 (36.9)		38 (40.4)	
Fever	0	55 (58.5)	535 (29.3)	<0.001	26 (27.7)	<0.001
	1	39 (41.5)	1,292 (70.7)		68 (72.3)	
Cough	0	43 (45.7)	582 (31.9)	0.007	38 (40.4)	0.556
	1	51 (54.3)	1,245 (68.1)		56 (59.6)	
Sputum production	0	73 (77.7)	1,543 (84.5)	0.107	79 (84.0)	0.354
	1	21 (22.3)	284 (15.5)		15 (16.0)	
Myalgia	0	85 (90.4)	1,349 (73.8)	<0.001	67 (71.3)	0.002
	1	9 (9.6)	478 (26.2)		27 (28.7)	
Fatigue	0	67 (71.3)	912 (49.9)	<0.001	45 (47.9)	0.002
	1	27 (28.7)	915 (50.1)		49 (52.1)	
Dyspnea	0	63 (67.0)	894 (48.9)	0.001	45 (47.9)	0.012
	1	31 (33.0)	933 (51.1)		49 (52.1)	
Diarrhea	0	94 (100.0)	1,720 (94.1)	0.029	90 (95.7)	0.121
	1		107 (5.9)		4 (4.3)	
Family history of tumor	0	93 (98.9)	1,773 (97.0)	0.519	89 (94.7)	0.211
	1	1 (1.1)	54 (3.0)		5 (5.3)	
Hypertension	0	68 (72.3)	1,376 (75.3)	0.597	65 (69.1)	0.748
	1	26 (27.7)	451 (24.7)		29 (30.9)	
Diabetes	0	83 (88.3)	1,607 (88.0)	0.949	83 (88.3)	1.000
	1	11 (11.7)	220 (12.0)		11 (11.7)	
Chronic liver disease	0	87 (92.6)	1,747 (95.6)	0.194	89 (94.7)	0.765
	1	7 (7.4)	80 (4.4)		5 (5.3)	
Malignancy	0	91 (96.8)	1,811 (99.1)	0.062	94 (100.0)	0.246
	1	3 (3.2)	16 (0.9)			
CKD	0	92 (97.9)	1,819 (99.6)	0.083	94 (100.0)	0.497
	1	2 (2.1)	8 (0.4)			
COPD	0	92 (97.9)	1,814 (99.3)	0.165	94 (100.0)	0.497
	1	2 (2.1)	13 (0.7)			
Severity of disease	Normal	62 (66.0)	1,218 (66.7)	0.933	64 (68.1)	0.597
	Severe	29 (30.9)	562 (30.8)		29 (30.9)	
	Critical	3 (3.2)	47 (2.6)		1 (1.1)	

*0, There is no such symptom or coexisting illness; 1, patients have this symptom or coexisting illness; CKD, chronic kidney disease; COPD, chronic obstructive pulmonary disease*.

### Clinical Features

The clinical features of the COVID-19 patients are shown in Table 1. Hypertension (27.7%) and diabetes (11.7%) were the most common coexisting illness in COVID-19 patients. Cough was the most common symptom in the antibody negative group. Other prevalent symptoms at the onset of illness of COVID-19 patients in the antibody-negative group included fever (41.5%), dyspnea (33.0%), fatigue (28.7%), expectoration (22.3%), and myalgia (9.6%), which were different between the antibody-negative and antibody-positive groups. The proportions of clinical classifications between the antibody-negative and antibody-positive groups (common cases, 66.0 vs. 68.1%; severe cases, 30.9 vs. 30.9%; critical cases, 3.2 vs. 1.1%) were not significantly different.

The laboratory findings revealed substantial differences between antibody-negative and antibody-positive patients (Table 2). Among patients with available data, white blood cell counts, neutrophils, C-reactive protein (CRP), procalcitonin (PCT), Interleukin-6 (IL-6), lactate dehydrogenase (LDH), creatine kinase, creatine kinase isoenzyme, urea nitrogen, and creatinine were significantly higher in antibody-negative patients than in antibody-positive patients (*P* < 0.005), with 143 (81.2%), 20 (18.3%), and 34 (44.2%) antibody-negative blood samples with increased CRP, PCT, IL-6 and 37 (19.9%) antibody-negative blood samples. In addition, those of the antibody-negative group were higher than those of the antibody-positive group (*P* < 0.05).

**Table 2:** Laboratory findings on the admission of patients with COVID-19.

		**Antibody-negative**	**Antibody-positive**	***P***	**Antibody-positive (after matching)**	***P***
*n*		94	1,827		94	
White-cell		6.6 [5.0, 9.1]	6.0 [4.8, 7.7]	0.003	6.0 [4.8, 7.6]	0.004
	<4	14 (8.5)	428 (10.2)	0.552	15 (6.8)	0.676
	≥4	151 (91.5)	3,757 (89.8)		205 (93.2)	
Neutrophils		4.3 [3.1, 6.6]	3.7 [2.8, 5.2]	<0.001	3.6 [2.8, 5.0]	<0.001
	<1.5	4 (2.2)	102 (2.1)	1.000	1 (0.4)	0.173
	≥1.5	182 (97.8)	4,657 (97.9)		238 (99.6)	
Lymphocyte		1.4 [0.9, 1.8]	1.4 [1.0, 1.8]	0.528	1.5 [1.0, 2.0]	0.110
	<0.8	37 (19.9)	734 (15.4)	0.122	28 (11.7)	0.029
	≥0.8	149 (80.1)	4,025 (84.6)		211 (88.3)
Platelets		209.0 [158.2, 262.8]	213.0 [169.0, 261.0]	0.138	215.5 [171.8, 265.2]	0.140
C-reactive protein		7.3 [1.3, 49.0]	2.4 [0.8, 9.2]	<0.001	1.7 [0.7, 6.8]	<0.001
	≤ 0.8	33 (18.8)	1,084 (24.1)	0.124	65 (29.1)	0.023
	>0.8	143 (81.2)	3,417 (75.9)		158 (70.9)	
PCT		0.1 [0.0, 0.2]	0.1 [0.0,0.1]	<0.001	0.0 [0.0, 0.1]	<0.001
	<0.5	89 (81.7)	2,374 (94.1)	<0.001	98 (100.0)	<0.001
	≥0.5	20 (18.3)	148 (5.9)			
IL-6		4.2 [1.5, 28.7]	2.2 [1.5, 7.6]	0.001	2.1 [1.5, 5.1]	0.001
	≤ 5.9	43 (55.8)	1,436 (71.9)	0.003	72 (81.8)	0.001
	>5.9	34 (44.2)	560 (28.1)		16 (18.2)	
ALT		17.4 [10.8, 30.4]	23.7 [15.1, 39.3]	<0.001	24.6 [13.8, 39.5]	<0.001
AST		19.7 [15.9, 29.4]	20.3 [15.9, 28.4]	0.898	19.9 [15.5, 28.1]	0.587
LDH		193.8 [154.9, 260.6]	187.3 [158.2, 232.1]	0.258	180.3 [157.5, 209.8]	0.006
CK		60.5 [40.5, 103.7]	42.2 [28.7, 62.4]	<0.001	37.3 [28.4, 61.5]	<0.001
CK-MB		10.3 [8.2, 14.5]	8.8 [7.0, 11.5]	<0.001	8.1 [6.4, 10.8]	<0.001
Albumin		36.7 [32.9, 40.6]	37.3 [34.0, 40.1]	0.308	37.5 [35.1, 40.0]	0.183
BUN		5.3 [4.0, 8.7]	4.9 [3.9, 6.2]	<0.001	5.1 [4.3, 6.4]	0.042
Creatinine		77.7 [60.6, 98.7]	63.6 [53.5, 75.9]	<0.001	69.8 [58.6, 81.7]	<0.001

*PCT, procalcitonin; IL-6, Interleukin- 6; ALT, alanine aminotransferase; AST, aspartate aminotransferase; LDH, lactate dehydrogenase; CK, creatine kinase; CK-MB, creatine kinase-MB; BUN, blood urea nitrogen*.

### Clinical Outcome

**Figure 1:**
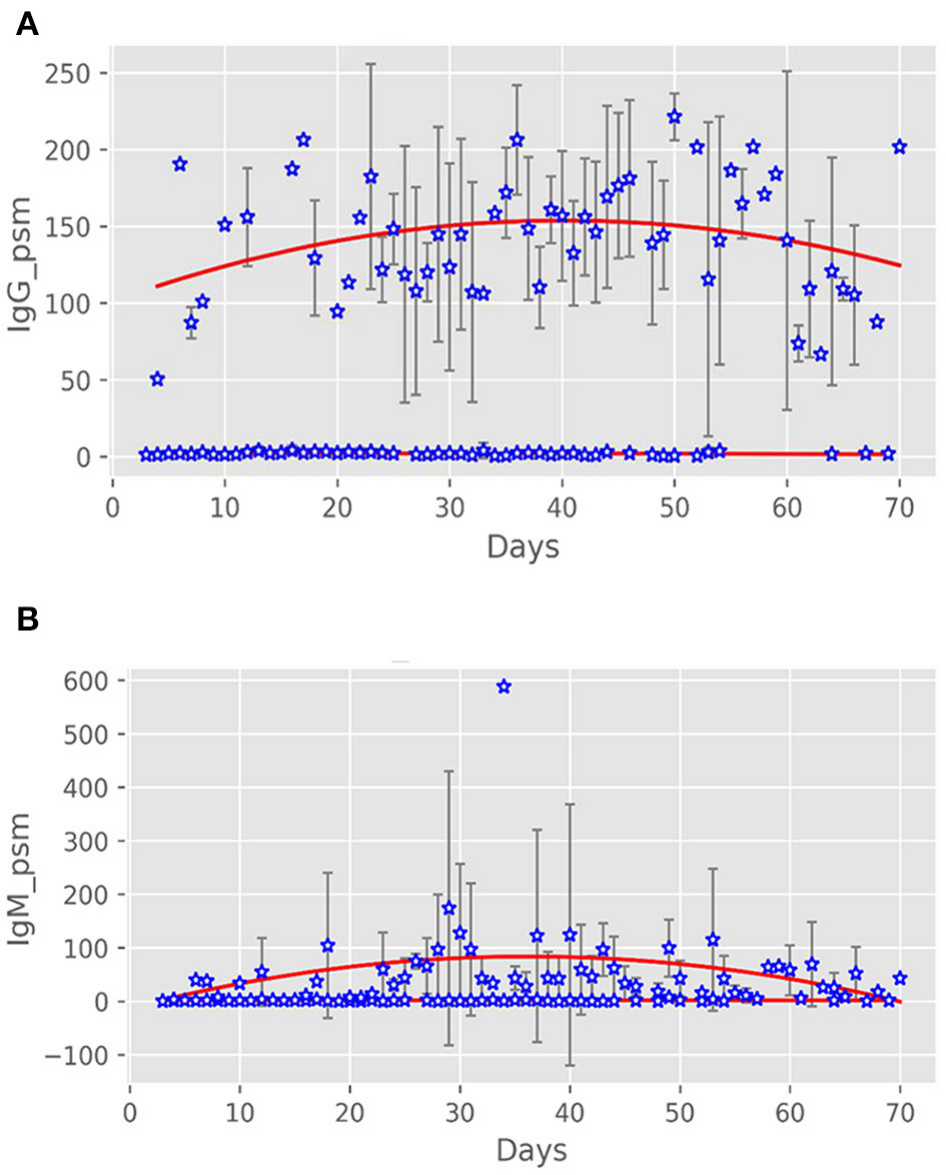
Antibody expression after onset of patients with COVID-19. **(A)** IgG expression after onset in the two groups of patients. **(B)** IgM expression after onset in the two groups of patients.

**Figure 2:**
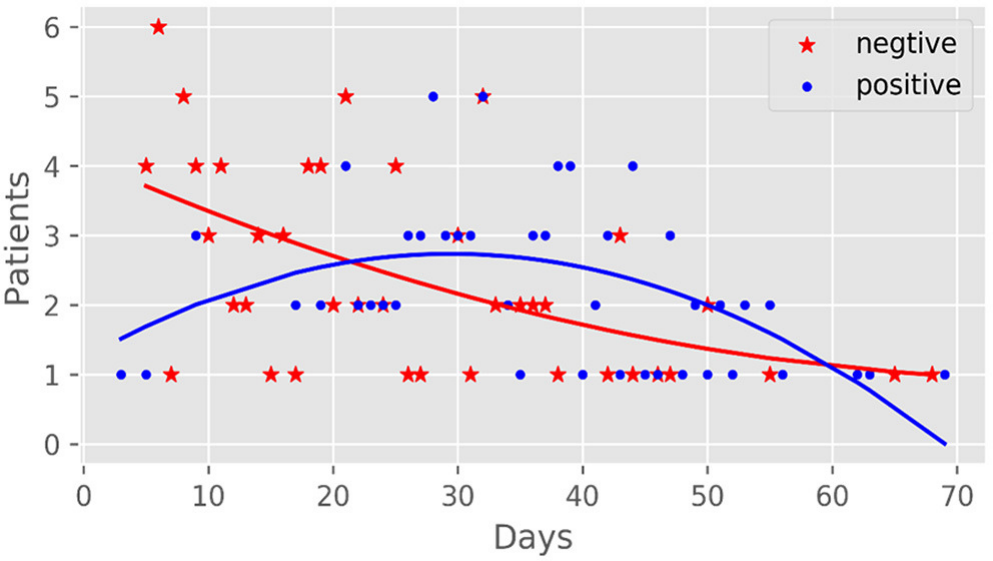
The Time of nucleic acid turning negative in patients with COVID-19.

There were 142 IgG detection results [155.51 (101.64, 185.83) AU/mL] and 141 IgM detection results [33.48 (11.51, 65.1) AU/mL] of 94 patients in the antibody-positive group (Figure 1). There was a significant correlation between antibody production and nucleic acid-negative conversion. The number of days of nucleic acid-negative conversion in the antibody-negative group was shorter than that in the antibody-positive group (*P* < 0.001) (Figure 2). The hospitalization time of antibody-negative patients was shorter than that of antibody-positive patients [8.0 (6.0, 10.0) vs. 13.0 (8.2, 23.0), *P* < 0.001] (Table 3). Three patients died during hospitalization in the antibody-negative group (3.2%) and no deaths occurred in the antibody-positive group. There were no significant differences between the two groups.

**Table 3:** Dynamic changes of antibodies in patients with COVID-19.

		**Antibody-negative**	**Antibody-positive (after matching)**	***P***
*n*		156	142	
IgM titer (AU/mL)		1.0 [0.6, 2.0]	33.5 [11.5, 65.1]	<0.001
IgM emergence after onset (d)		20.0 [13.0, 31.2]	40.0 [31.0, 48.8]	<0.001
IgG titer (AU/mL)		1.7 [0.9, 2.6]	155.5 [101.6, 185.8]	<0.001
IgG emergence after onset (d)		20.0 [13.0, 31.2]	40.0 [31.0, 48.8]	<0.001
The Time of nucleic acid turning negative (d)		19.0 [10.0, 30.0]	37.0 [26.0, 46.8]	<0.001
The time of hospitalization (d)		8.0 [6.0, 10.0]	13.0 [8.2, 23.0]	<0.001
Outcome	Survivors	91 (96.8)	94 (100.0)	0.246
	Non-survivors	3 (3.2)		

## Discussion

In this study, we identified no significant difference in sex, age, non-survivors, and clinical classification between the antibody-negative and antibody-positive groups. Compared with antibody-positive patients, antibody-negative patients had lower rates of fever, myalgia, fatigue, and dyspnea. In terms of laboratory findings, white blood cells, neutrophils, CRP, PCT, IL-6, LDH, and creatine kinase levels were significantly higher in antibody-negative patients than in antibody-positive patients. Significantly, 143 (81.2%), 20 (18.3%), and 34 (44.2%) antibody-negative blood samples had increased levels of CRP, PCT, IL-6, and 37 (19.9%) antibody-negative blood samples had lymphocytopenia. Numerous studies have shown that when bacteria, viruses, and other microorganisms invade the human body, the body removes the harmful microorganisms through innate and adaptive immunity. It produces immunoglobulins that can bind to target antigens through adaptive immunity and can accurately and efficiently remove harmful substances. However, some COVID-19 patients can still clear the virus without producing specific antibodies. The current study attempted to address the characteristics of these patients, the reliance on innate immunity to remove the virus, and the relationship between the novel coronavirus and human immune response. We analyzed the clinical characteristics and outcomes of COVID-19 patients without specific antibodies against SARS-CoV-2 using electronic medical records.

The common symptoms in antibody-negative patients included in this study were cough, fever, dyspnea, fatigue expectoration, and myalgia. The incidence rate was lower than that in the antibody-positive group and other related studies (7, 8). This may be related to the inhibition of viral activity and the weakening of viral pathogenicity by an inflammatory reaction *in vivo*. The types of coexisting illnesses and the proportion of severe/critical cases in the antibody-negative group were similar to those in the antibody-positive group and other studies (9, 10), suggesting that the antibody non-responders conformed with the overall distribution characteristics of COVID-19 patients. Wang et al. studied the relationship between antibody and virus clearance time in 26 patients with COVID-19 and found that the early production of antibodies does not necessitate the early elimination of this virus. They did not observe a correlation between early adaptive immune responses and better clinical outcomes. One patient did not produce specific antibodies against SARS-CoV-2 within 66 days of observation. Eventually, the nucleic acid test for SARS-CoV-2 turned negative, revealing that some individuals may not produce antibodies after being infected with SARS-CoV-2 (11). Tan et al. Divided the antibody responses of study participants into three groups: strong response, weak response, and no response; that is, no specific immune response was observed, but the virus was still cleared. They suggested that strong antibody responses were associated with disease severity, while weak antibody responses were associated with virus clearance. However, in this study, the definition of no response patients is that as long as patients do not produce IgM or IgG specific antibodies, it does not apply to patients who do not produce IgM and IgG specific antibodies simultaneously (12). Another study found negative serum antibody test results 13% of patients (13). In our study, 94 of the 1,921 patients did not produce specific antibodies. However, viral nucleic acids were still cleared during hospitalization, which may prove that the innate immune system is involved in clearing SARS-CoV-2.

CRP is a non-specific inflammatory marker and an acute-phase reaction protein that can activate, complement and strengthen phagocyte function, and remove pathogenic microorganisms invading the body and tissue cells that are damaged, necrotic, and apoptotic. IL-6 is also a non-specific indicator of inflammation. Inflammatory cytokines produced by various cells after inflammatory stimulation are critical components of the inflammatory response and can induce an increase in CRP and PCT at 2 and 6 h after infection, respectively. PCT reflects systemic inflammation, which increases slightly when the virus has infected. When the pathogen invades the body, the human immune system enters the immediate innate immune response stage. Neutrophils are the central effector cells, and the total number of leukocytes and neutrophils increases significantly. In the early stage of the innate immune response, activated neutrophils produce pro-inflammatory cytokines such as IL-6, whereas hepatocytes produce a series of acute-phase proteins after being stimulated by pro-inflammatory cytokines such as interleukin-1, of which CRP is the most significant. Zhu et al. found that interferon-stimulated genes (such as ISG15, IFI44L, and MX1) in the peripheral blood immune cells of patients with COVID-19 were significantly upregulated, which confirmed that the innate immune response was significantly activated in patients with COVID-19. Meanwhile, the concentration of IL-6 in patients with COVID-19 was significantly higher than that in the general population (14). The total number of white blood cells, neutrophils, CRP, IL-6, PCT, and other inflammatory cells were higher in the antibody-negative group in this study. It was confirmed that after SARS-CoV-2 entered the body, the innate immune response of this population is rapid, intense, and cleared the virus quickly. Other studies have found that pathogenic T cells are activated rapidly to produce GM-CSF and IL-6, wherein GM-CSF further activates CD14+ and CD16+ inflammatory monocytes to produce more IL-6 and other inflammatory cytokines, resulting in an inflammatory storm (15). Innate immunity and T cell-mediated immune damage might be caused by pro-inflammatory factor-induced inflammation, which plays a vital role in the occurrence and development of COVID-19. The number of lymphocyte-decreased patients in the antibody-negative group was greater than that in the antibody-positive group, suggesting that the inhibition of the virus on lymphocytes (16) weakened the adaptive immune response and delayed the production of specific neutralizing antibodies by B lymphocytes. Strong inflammatory reactions spread throughout the body, involving many target organs, such as the liver and kidney, resulting in a significant increase in LDH, creatine kinase, creatine kinase isoenzyme, urea nitrogen, and creatinine. These results are significant, indicating that the neutrophil/lymphocyte ratio (NLR) in most antibody-negative patients is higher than that in antibody-positive patients. Considering that the neutrophil/lymphocyte ratio (NLR) is a strong indicator of innate immunity, the other hand, it shows that antibody-negative patients mainly rely on congenital immune reaction to clear the virus (17, 18).

Gallais et al. observed that individuals exposed to SARS-CoV-2 can induce virus-specific T cell response without seroconversion. However, the study included only nine confirmed patients and eight suspected patients, and our results enriched such results in the number of samples (19). Other studies have confirmed that innate immunity can effectively clear SARS-CoV-2 during mild and short-term infection (20). Therefore, we believe that the main reason why 94 patients do not produce specific antibodies is that there are SARS-CoV-2 specific humoral immunity and innate immunity in COVID-19 patients. However, SARS-CoV-2 does not induce a humoral immune response in some patients with strong innate and cellular immunity (21). In addition, there may be many other possibilities that these patients do not produce specific antibodies. First, there may have been a mutation in SARS-CoV-2. Infected patients may have produced more specific antibodies, so we could not detect them by routine testing. Secondly, the negative serum antibody may be caused by some immune deficiency in the patients. It has been reported that the antibody response to SARS-CoV-2 is delayed in patients infected with human immunodeficiency virus (22), so there may be some particular patients who cannot produce antibodies response to SARS-CoV-2. Lusida et al. found that 32 of 45 infected patients always showed serum negative antibodies, and the 32 study participants were all staff of medical research institutions (23). Another study reported that the positive serum rate was closely related to age and symptoms and decreased in the elderly; there were fewer social contacts in this age group than that among active young people. In addition, more stringent preventive behaviors have been taken in high-risk groups to limit exposure to the virus (24). Therefore, the excellent COVID-19 education, stricter infection prevention, control measures, and less contact with society may explain why these participants remain seronegative. These guesses need to be confirmed by further research.

We found no difference in the proportion of clinical classifications between antibody-negative and antibody-positive groups. Compared with antibody-positive patients, antibody-negative patients had lower rates of fever, myalgia, fatigue, and dyspnea. The nucleic acid negative time and length of hospital stay in the antibody-negative group were shorter, which means that our study emphasizes the possible correlation between symptoms and serum antibodies. The severity of coronavirus pneumonia does not seem to affect humoral immunity. Previous data show that severe COVID-19 patients have stronger serological responses than mild patients, these patients have earlier seroconversion and higher IgG antibody levels, while mild SARS patients have lower antibody titers (25, 26). Chia et al. found that the duration of IgG antibody levels was associated with the severity of the disease, and patients with mild conditions seemed to exhibit faster IgG antibody regression (27). In another study, IgM and IgG responses in patients with moderate or severe disease were significantly higher than those in patients with mild or asymptomatic infection, and IgM and IgG concentrations in all groups were significantly higher than those in the control group (28). However, the populations included in these studies were actively mobilizing specific antibody responses to clear the virus. The results of these studies may not apply to those who do not produce antibodies.

In this study, we focused on a group of patients diagnosed with COVID-19 by RT-PCR with no detected serum antibodies. Of the 1,921 patients enrolled by Huoshenshan Hospital, ~5% were seronegative throughout the course of the disease, which is much smaller than the results of existing studies. In the study by Sina et al., undetectable humoral reactions were observed in 17% of the patients (29). Another study reported that 10% of the participants had no antibodies (12); in a study in Indonesia, 32 of the 45 diagnosed patients remained antibody-negative (23). These results indicate the potential diagnostic value of serum antibodies against SARS-CoV-2 infection. Some patients remain antibody-negative throughout the disease course after the diagnosis of COVID-19, which indicates that antibody detection is unsuitable as a serological marker for diagnosing early COVID-19 infection in this part of the population.

The virus clearance time in antibody-negative patients was significantly shorter than that in antibody-positive patients. The shedding time of viruses in other related studies varied greatly from 11 to 20 days (9, 30). After eliminating confounding factors, such as age and coexisting illness, the hospitalization time of the antibody-negative group was significantly shorter than that of the antibody-positive group, which confirmed that the rapid clearance of the virus was related to the short hospitalization time. Of the deaths, 3/94 (3.2%) were in critically ill patients, and the mortality rate was lower than the current results of related studies (8, 31). Activation of the innate immune response is necessary to eliminate the invading virus effectively, but its abnormal activation and excessive production of pro-inflammatory cytokines may cause damage to the host tissue. Galloway et al. scored COVID-19 patients on 12 items, wherein the higher the score, the greater the risk of CCU or death, including neutrophil count >8.0 × 10^9^/L, and CRP >40 mg/L. Another study listed leukocytes >10 × 10^9^/L and neutrophils >7.5 × 10^9^/L as risk factors for adverse outcomes (32). However, there was no significant difference in mortality between the two groups in our study. Whether these studies suggest that excessive inflammation is related to death in critically ill patients warrant further investigation.

The long-term humoral response to SARS-CoV-2 infection is key to assessing population immunity, potential risk of reinfection, and vaccine response. Recent studies have confirmed that antibodies still play a protective role 7 months after the initial infection (33), and the effective rate of preventing reinfection is ~95% (34). A study of 12,541 health care workers showed that the presence of anti-spike or anti-nucleocapsid IgG antibodies was significantly associated with a significantly reduced risk of SARS-CoV-2 reinfection over the next 6 months (35). Another study reported a higher percentage of new infections among serum-negative participants compared with only one reinfection among 47 serum-positive participants (36). This suggests that patients who do not produce antibodies or exhibit milder symptoms at the time of initial infection may have a higher chance of reinfection, emphasizing the need for vaccination. However, compared to the general population, patients whose antibodies are always negative after infection may have a high non-response rate, low immunogenicity, and low effectiveness of the COVID-19 vaccine. We need to consider whether it is necessary to increase the scope and dose of vaccinations. Further research is needed to significantly improve long-term vaccination strategies. Further research is needed to significantly improve long-term vaccination strategies.

Our study had some notable limitations. First, because of its retrospective nature, more detailed laboratory testing of immune cell inflammatory factors was lacking, such as T cell count, tumor necrosis factor, and various types of interleukins, to more accurately judge the degree of inflammatory response in COVID-19 patients. Second, there was a significant difference between the antibody-negative and antibody-positive groups. Although we used 1:1 PSM, bias inevitably affected our assessment.

## Conclusion

Some COVID-19 patients without specific antibodies exhibited mild symptoms. However, the inflammatory reaction caused by innate clinical immunity was more intense than that caused by antibodies, as seen through the faster virus clearance. Non-specific immune responses played an essential role in virus clearance. There was no direct correlation between excessive inflammatory response and adverse outcomes of patients. The risk of reinfection and vaccination strategies for antibody-negative patients need further research.

## Data Availability Statement

The data that support the findings of this study are available from the Huoshenshan Hospital and Chinese PLA General Hospital, but restrictions apply to the availability of these data, which were used under license for the current study, and so are not publicly available. Data are however available from the authors upon reasonable request and with permission of the Huoshenshan Hospital and Chinese PLA General Hospital. Requests to access these datasets should be directed to XG, gxz301@126.com.

## Ethics Statement

The studies involving human participants were reviewed and approved by Ethical Review of Scientific Research Projects of the Medical Ethics Committee of the Chinese PLA General Hospital. Written informed consent for participation was not required for this study in accordance with the national legislation and the institutional requirements.

## Author Contributions

JD and CL wrote the first draft of this manuscript. HG and BW handled laboratory logistics and generated data. ZW analyzed and summarized the data. ZW and LM provided input on assay design and interpretation of results. BW, HZ, and MS provided inputs for data collection and interpretation of the results. XG developed the project concept and guided laboratory work. All authors reviewed and edited all the sections of the article.

## Funding

This work was supported by the Ministry of Industry and Information Technology of China (2020-0103-3-1).

## Conflict of Interest

The authors declare that the research was conducted in the absence of any commercial or financial relationships that could be construed as a potential conflict of interest.

## Publisher's Note

All claims expressed in this article are solely those of the authors and do not necessarily represent those of their affiliated organizations, or those of the publisher, the editors and the reviewers. Any product that may be evaluated in this article, or claim that may be made by its manufacturer, is not guaranteed or endorsed by the publisher.
